# Supplementary material for: FORGe: prioritizing variants for graph genomes
Source: Genome Biol. 2018 Dec 17;19:220. doi: 10.1186/s13059-018-1595-x (PMC6296055; doi:10.1186/s13059-018-1595-x)
Supplement: Supplementary file 1 — Supplementary information. Contains Notes S1–S7 and Figures S1–S9. (PDF 620 kb) [file 13059_2018_1595_MOESM1_ESM.pdf]

Supplement for: FORGe: prioritizing variants for graph genomes

Jacob Pritt[1, 2], Nae-Chyun Chen[1, 2], and Ben Langmead[1, 2]

[1]Department of Computer Science, Johns Hopkins University
[2]Center for Computational Biology, Johns Hopkins University

November 23, 2018

Note S1 Effect of Variant Density on Graph Size

Note S1.1 Sources of blowup

Graph genome methods can suffer from exponential blowup caused by densely-packed variants. Without information about which alleles co-occur on haplotypes, all possible combinations must be considered. When variants are spread out, the problem is mitigated by the intermediate unambiguous sequence. But groups of densely-packed variants can lead to increases in the size of the graph genome representation, the size of the genome index, and the amount of time required to align reads. Exactly what kind of penalty is incurred depends on the method. We discuss a few concrete examples that convey the various ways blowup can manifest.

ERG. As described in the main text, the Enhanced Reference Genome (ERG) method [1] builds an augmented reference genome by adding *enhanced segments*: reference substrings that include alternate alleles and flanking context determined by the read length. When k variants co-occur in a window, $2^k - 1$ enhanced segments are added to cover all combinations of ALT and REF alleles (see Fig S1). This increases the size of the reference genome representation and accompanying index.

GCSA. GCSA [2] builds an FM-index-like data structure over a directed acyclic graph (DAG) representation of reference genome and its variants. The index requires that nodes of the DAG be "prefix-sorted." The prefix of a node is the longest common prefix of all the paths originating at the node and ending at the end of the sequence; these are essentially

suffixes with the added complication that a single node can have many suffixes since many allelic assignments are possible downstream. The DAG that arises naturally from a multiple alignment of the reference sequences does not generally have the prefix-sorted property; rather, the property must be added through a series of transformations that add nodes to the graph. This is the "path doubling" process described in the GCSA study, and it is after this step that "the prefix-range-sorted automaton (DAG) can be exponentially larger than the finite automaton from which it is built." The blowup is hinted at in the transformation shown in Figures 4 and 5 of that study, but the clearer demonstration is in the empirical results presented in its Table 2, which shows that the size of the index varies widely from chromosome to chromosome, with some relatively short chromosomes having unexpectedly large indexes (e.g. Chr 17) and others failing to build at all due to memory exhaustion (e.g. Chr 18) [2].

BWBBLE. BWBBLE [3] takes a wildcard-based approach, replacing SNVs with the IUPAC code corresponding to the REF and ALT alleles together. For instance, a position where `C` is the reference allele and `T` is the alternate allele would be replaced by the IUPAC code `Y`, meaning `C`-or-`T`. This linear sequence with IUPAC codes is then indexed with a typical FM Index. The FM Index itself, however, does not respect the meaning of the IUPAC codes. Instead, BWBBLE also introduced a new method for querying the index that is capable of matching unambiguous bases to IUPAC codes. To accomplish this, though, the algorithm must try all possible legal substitutions of a concrete base for an IUPAC code. Thus, in the case of BWBBLE, the most direct impact of having many variants (IUPACs) near each other in the reference is the induce an exponential amount of backtracking in the alignment algorithm.

BWBBLE's strategy for including indels in the reference does not use IUPAC codes. Rather, it is essentially the same as the ERG method described above. In that case, the most direct effect of the blowup is to increase the size of the reference representation and index.

Note S1.2 Sources of alignment errors

The read aligner's task is to assign each read to its true point of origin with respect to the reference. When the reference is ambiguous — e.g. contains interspersed repeats, long stretches of low-complexity sequence, or segmental duplications — the read aligner is more likely to place the read incorrectly. For example, if a read that aligns with equal alignment score to ten copies of a repetitive element, the aligner has no basis for choosing one over the others and has no better than a one-in-ten chance of choosing correctly.

Adding variants to the reference has advantages and drawbacks, with the chief drawback being that the reference genome becomes more ambiguous, leading to more repetitive alignments. This can eventually lead to situations where reads that aligned correctly

to genome G will align incorrectly to genome G', where G' contains a superset of the variants contained in G. This is illustrated in Fig S2.

Note S1.3 Strategies for mitigating blowup

The manuscript discusses two methods for mitigating blowup from densely packed variants. One is to disallow arbitrary patterns of recombination between variants and instead allow only combinations corresponding to haplotypes in a reference panel, such as the 1000 Genomes Project Phase-3 call set [4]. Considered in the context of the ERG method [1] and its use of "enhanced segments," this limits the maximum possible number of segments covering a genomic position to be at most the number of reference haplotypes. Contrast this with $2^k - 1$ segments, which is the number of segments required when k variants co-exist in a single window. Though FORGe does not make use of this strategy, a strategy like this (though with somewhat more arbitrarily defined haplotype blocks) is used by HISAT2's [5] default pruning technique.

Another method is the one explored in this manuscript: to prioritize and select variants so as to avoid dense clusters. This is accomplished through a combination of our blowup avoidance strategies (in the *Pop Cov+* and *Hybrid+* models) and our selection of only the top-ranked variants according to the models. This method is applicable even when a panel of reference haplotypes is not available.

Note S2 Simulating from NA12878

We downloaded the GRCh37 genome FASTA as well as the phase 3 variant call set in VCF format from the 1000 Genomes Project [4]:

```
ftp.1000genomes.ebi.ac.uk/vol1/ftp/release/20130502/
```

We selected all variants that appeared in either of NA12878's haplotypes and created a customized FASTA for each haplotype.

We generated a custom genome for NA12878 using the `update_genome.py` script in the `scripts` subdirectory of the `FORGe-experiments` repository. `update_genome.py` takes as input a linear reference genome, a VCF containing variants, and the individual name (e.g. "NA12878") for which we wish to construct a custom genome.

We used Mason 0.1.2 to simulate 10 million 100bp unpaired reads from each haplotype using this command:

```
mason illumina -N 10000000 -n 100 -hs 0 -hi 0 -hn 1 -mp -i -sq
```

Scripts used to perform these steps are described in more detail in

```
https://github.com/langmead-lab/FORGe-experiments/tree/v1.0
```

Note S3 Software implementing the Enhanced Reference Genome

FORGe implements the method described in the Enhanced Reference Genome (ERG) study [1] for iterating over all added segments, but with two modifications:

- FORGe considers all alternate alleles, even for variants with 2 or 3 alternate alleles
- FORGe does not implement an upper limit on the number of variants per window

Each segment is appended to the end of the genome FASTA file as a new sequence, with the sequence name indicating the position in the linear reference genome where the all-reference-allele version of the sequence begins. After aligning reads to the augmented reference, a postprocessing script maps the alignment coordinates back to the appropriate location on the linear reference.

Our implementation of the ERG method is located in the `src` subdirectory of the `FORGe` source repository on GitHub:

```
https://github.com/langmead-lab/FORGe/tree/v1.0
```

Note S4 Generating variant set

Using the same variant VCF file discussed in the previous Note, we filtered the columns to remove individuals NA12877–NA12893, who are all relatives of NA12878. We then processed every SNV in the file as follows: any variant that appeared in at least one of the remaining columns was added to our variant set, with alternate allele frequency equal to the number of total appearances of that allele in all haplotypes divided by the total number of haplotypes.

For more details see script `vcf_to_1ksnp.py` and the FORGe README in

```
https://github.com/langmead-lab/FORGe/tree/v1.0
```

Note S5 Time and space limitations of HISAT2 index building

HISAT2 suffers from memory blowup during index building when the reference genome or variant set are complex. Specifically, even with 1 TB of RAM HISAT2 was unable to build a graph-genome index for the top-scoring 10% of SNVs from the full genome, chosen according to the population coverage strategy. After incorporating the blowup avoidance re-ranking strategy into FORGe, HISAT2 was able to construct the index for up to 80% of SNVs with less than 210 GB of RAM. For 90% of variants or higher, index construction still exhausted memory and failed.

Note S6 Full genome ranking strategy limitations

The *Hybrid* ranking strategy requires that we iterate over all k-mers (elsewhere called s-mers) in $G*$ in order to count each k-mer's frequency. In the released version of FORGe, this counting is accomplished using the Jellyfish k-mer counting tool. Since the number of k-mers in the augmented genome can be very large – tens of billions of distinct k-mers in our experiments – the time and memory required to perform this count makes the *Hybrid* approach far less practical than *Pop Cov* for large references.

That said, we are also releasing a version of FORGe that uses the KMC3 k-mer counter instead of Jellyfish. This substitution, together with a reengineering of the algorithm to perform updates and queries in a "batch" fashion, make FORGe's *Hybrid* model much more scalable to large genomes and variant sets. We were able to run this version on the whole human genome, giving the full set of 1000 Genomes Project phase-3 callset variants as input. This version is located in the `kmc` branch of the FORGe repository.

This new version of the code is not yet as feature-complete as the standard version of FORGe. It does not yet respect the `--phasing` option, and its use of KMC3 also means that its k-mer counts saturate at 255; i.e. counts higher than 255 are rounded down to 255. Expanding on this mode and merging it with FORGe's default mode remains important future work.

Note S7 HLA typing experiment

We sought to measure how HLA typing recall varies with the fraction of variants included in the reference. We used the same NA12878 "Platinum reads" (ERR194147) evaluated in the results section. Note that these are paired-end reads, but we used only the first end of the pair and performed unpaired alignment. For each BAM file — one per variant inclusion fraction: 0%, 2%, 4%, 6%, 8%, 10%, 15%, and 20% up to 100% in 10 point increments — we extracted the alignments from the MHC region and converted the alignments back into FASTQ reads. We then provided these reads to the Kourami [6] HLA typing tool. Kourami realigns the reads using BWA-MEM [7] to its internal HLA allele panel. These alignments, as well as the reads that BWA-MEM failed to align, are then used by Kourami to call HLA alleles. There are 12 possible calls (2 haplotypes for 6 HLA genes) but Kourami makes calls only where there is sufficient evidence.

We compared the HLA calling results are compared to the validated types in the Population Reference Graph study [8]. Four-digit calling accuracy is measured in this experiment because of the limitation of validation results, but our workflow is able to type HLA alleles to the G group level. When the top-scoring 10% of variants were included in the reference, 25,347 more reads (1.02% increase) mapped to the MHC region and HLA allele recall increased from 50% (6 out of 12 alleles correctly called) to 91.7% (11 out of 12). HLA typing recall remained at 91.7% at higher variant inclusion fractions. Average cov-

erage did not substantially increase after 10% of variants were included, and decreased somewhat after 20%.

The commands used to perform the experiment are shown below.

```
# extract reads in HLA region
samtools view -b forge.bam 6:28477797-33448354 > forge_mhc.bam
# convert to fq
samtools bam2fq forge_mhc.bam > forge_mhc.fq
# align reads to kourami_db (pre-processing step required by kourami)
bwa mem kourami_db forge_mhc.fq > forge_mhc-kourami_db.sam
# convert sam to bam
samtools view -b forge_mhc-kourami_db.sam > forge_mhc-kourami_db.bam
# run kourami
java -jar Kourami.jar -d kourami_db -o output forge_mhc-kourami_db.bam
```

We investigated why our method called only 11 of 12 HLA alleles correctly in NA12878. The failed call was for an allele of the DQB1 gene. The true alleles for this (diploid) site in NA12878 are DQB1*02:01 and DQB1*05:01. When we included 6% or less of the FORGe-selected variants in the graph, Kourami had insufficient coverage to make a call for DQB1. When we included 8% or more, Kourami consistently called the DQB1*05:01 allele for both haplotypes. The reference genome itself has allele DQB1*06:02.

We hypothesized that the missed call was due to our failing to align many reads from the DQB1*02:01 allele, even after including of the 1000 Genomes Project variants. We examined how many of the ALT sites in DQB1*05:01 (which we call correctly) and DQB1*02:01 (which we fail to call) were contained in the 1000 Genomes Project phase 3 callset. We found that DQB1*05:01 had ALT 17 sites of which 11 (64.71%) were in the 1000 Genomes Project callset, whereas DQB1*02:01 had 32 ALT sites of which 13 (40.63%) were in the callset. Thus, we contend that Kourami failed to call DQB1*02:01 because, even after including the 1000 Genomes variants, there were still enough non-reference alleles in DQB1*02:01 for those reads to fail to align.

It is important to note that the standard Kourami method begins by aligning sequencing reads to the GRCh38 version of the human genome reference, including the so-called "ALT loci," which are alternate assemblies of some hypervariable portions of the genome including the HLA genes. Since the Kourami study was able to call all 12 alleles correctly for NA12878 on similar data, this highlights the fact that the relatively small-scale variants called in the 1000 Genomes Project, while helpful, may not be sufficient for downstream analyses. There are still advantages to aligning to entirely separate assemblies, such as the ALT loci, especially in hypervariable regions.

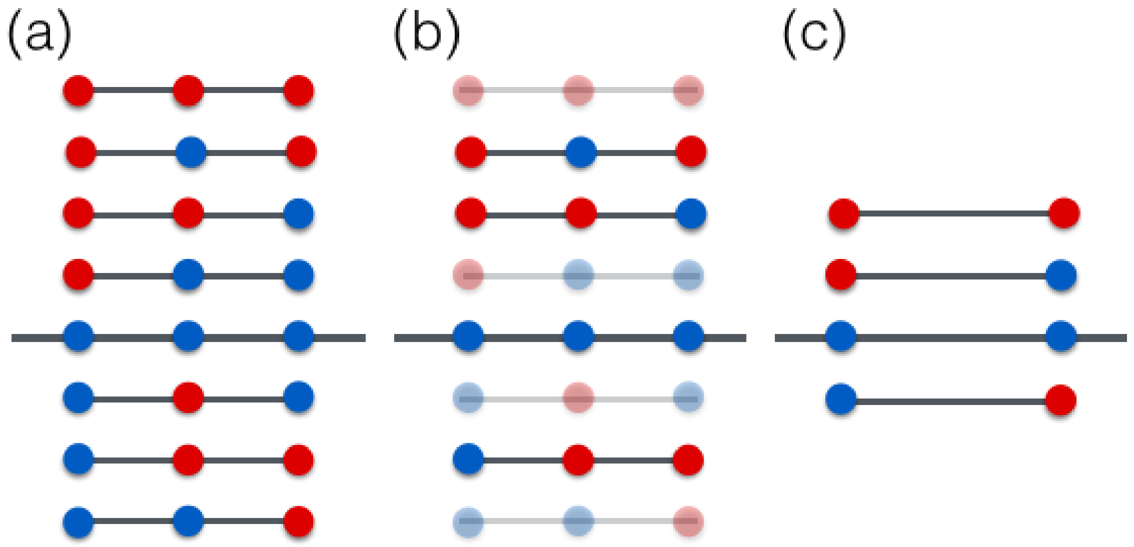

Fig S1: (a) In the ERG method, k closely-placed variants result in $2^k - 1$ enhanced segments being added to the graph genome. To mitigate this blowup, we might (b) limit the added contigs to correspond only to those with combinations of alleles present in a panel of reference haplotypes (c) filtering the initial set of variants.

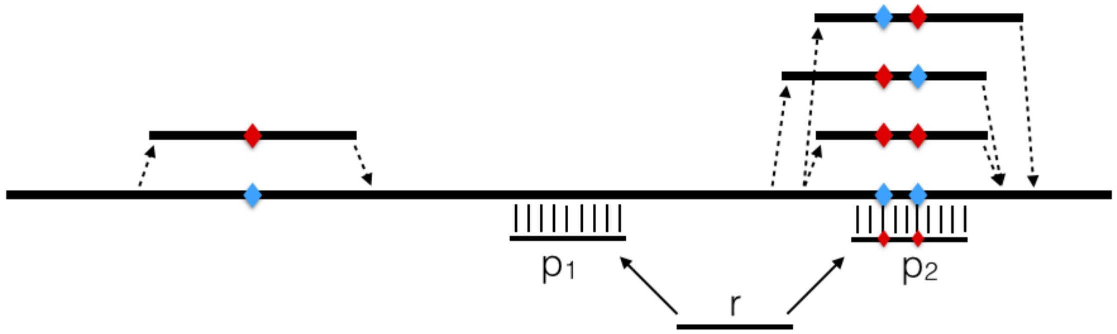

Fig S2: Example where adding variants creates more ambiguity in the reference. The horizontal line represents the reference genome. Blue diamonds represent reference alleles and red diamonds represent alternate alleles. The shorter lines represent enhanced segments, per the ERG method. Read r maps uniquely to the linear reference genome at position p_1. The addition of the group of variants on the right creates a new perfect match for r at position p_2, reducing overall uniqueness.

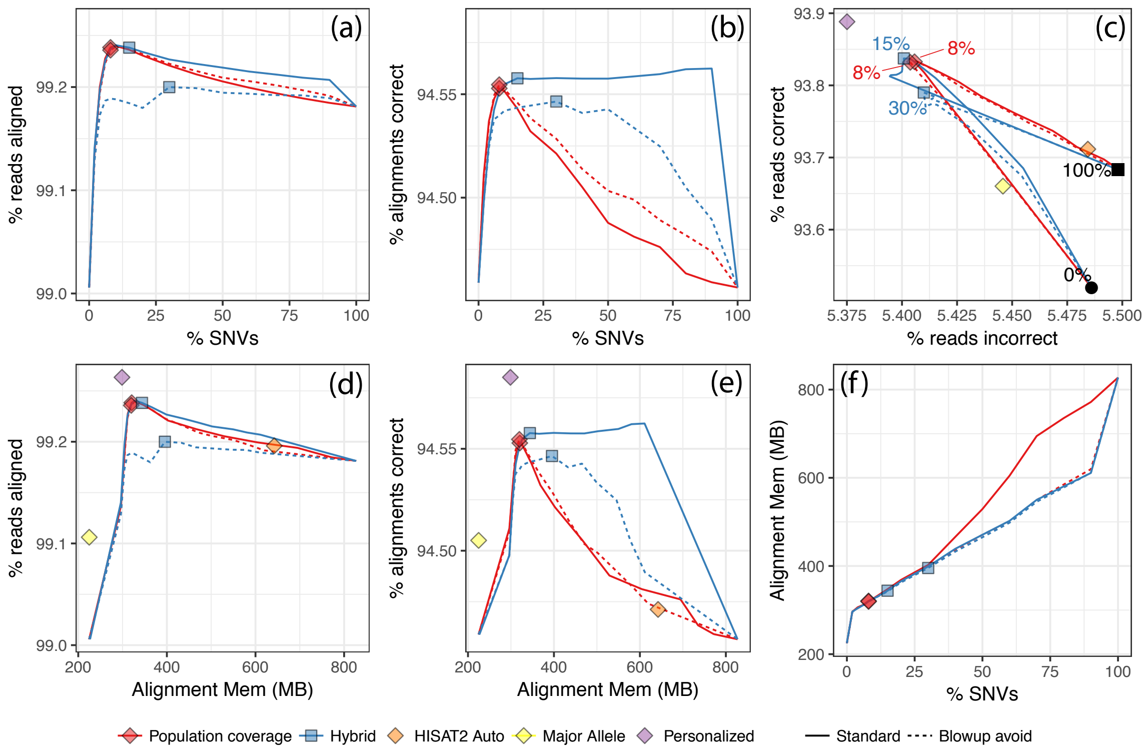

Fig S3: Accuracy for paired-end reads simulated from chromosome 9 of NA12878. All other parameters were the same as for Figure 1. Panels and plots have the same meaning as for Figure 1 except that the random ranking is omitted here.

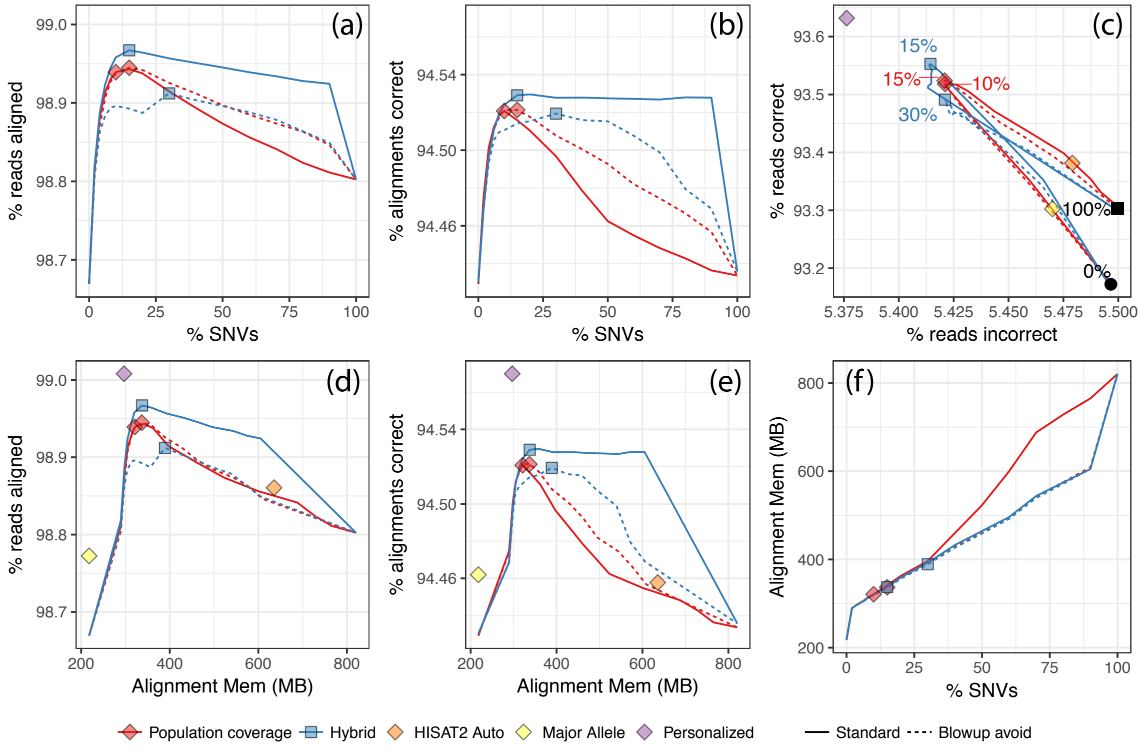

Fig S4: Accuracy for reads simulated from chromosome 9 of YRI individual NA19238. The initial variant set contained all 1000 Genomes SNPs in chromosome 9, after removing NA19238 and direct relatives. Panels and plots have the same meaning as for Figure 1 except that the random ranking is omitted here.

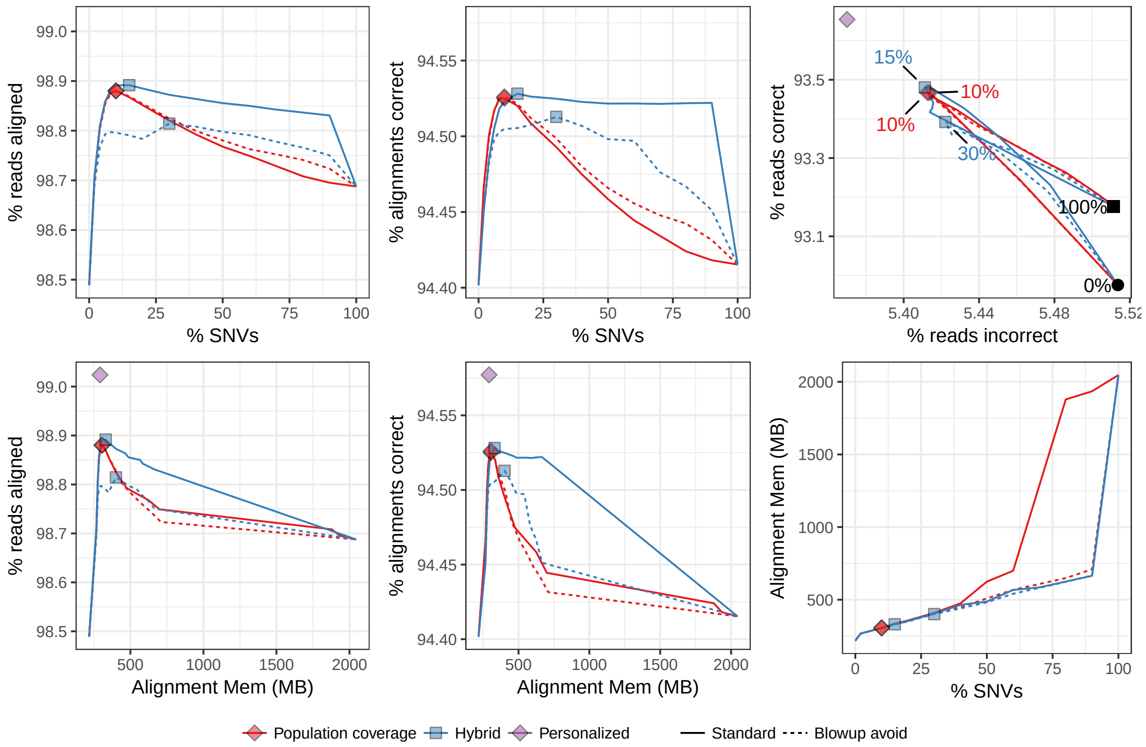

Fig S5: Accuracy when both SNVs and indels are modeled and included in the augmented reference. All other experimental parameters were the same as for Figure 1. Panels and plots have the same meaning as for Figure 1 except that the random ranking is omitted here.

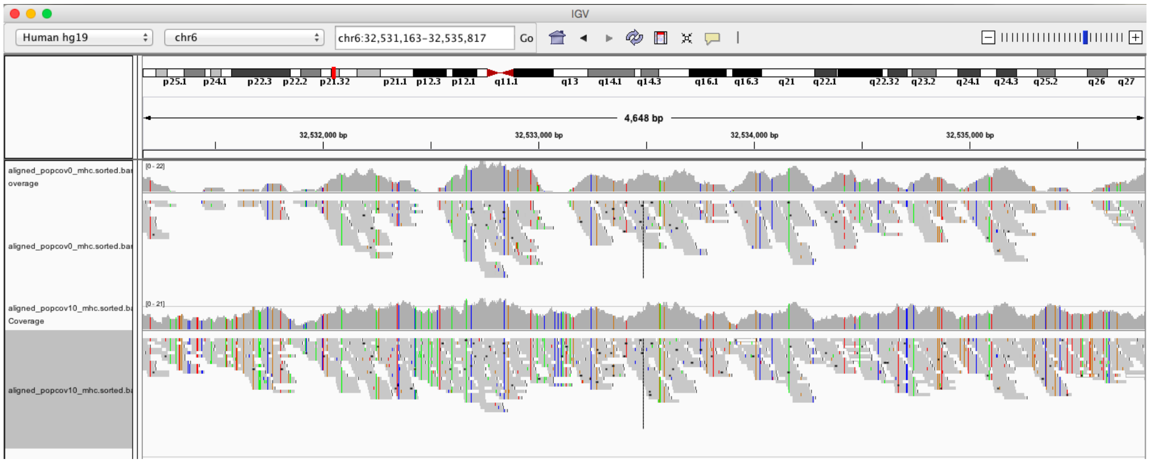

Fig S6: Substantial improvements in alignment coverage in the MHC region when variants are included in the graph genome. Simulated reads from chromosome 6 of NA12878 were aligned against the linear reference genome (top) and the graph genome including the top-scoring 10% of SNVs ranked according to the *Pop Cov* model (bottom). Visible in the pileup are are multiple places where linear-genome coverage decreases to 0, but graph-genome coverage remains high, because the graph genome includes some or all of the variants in the dense clusters.

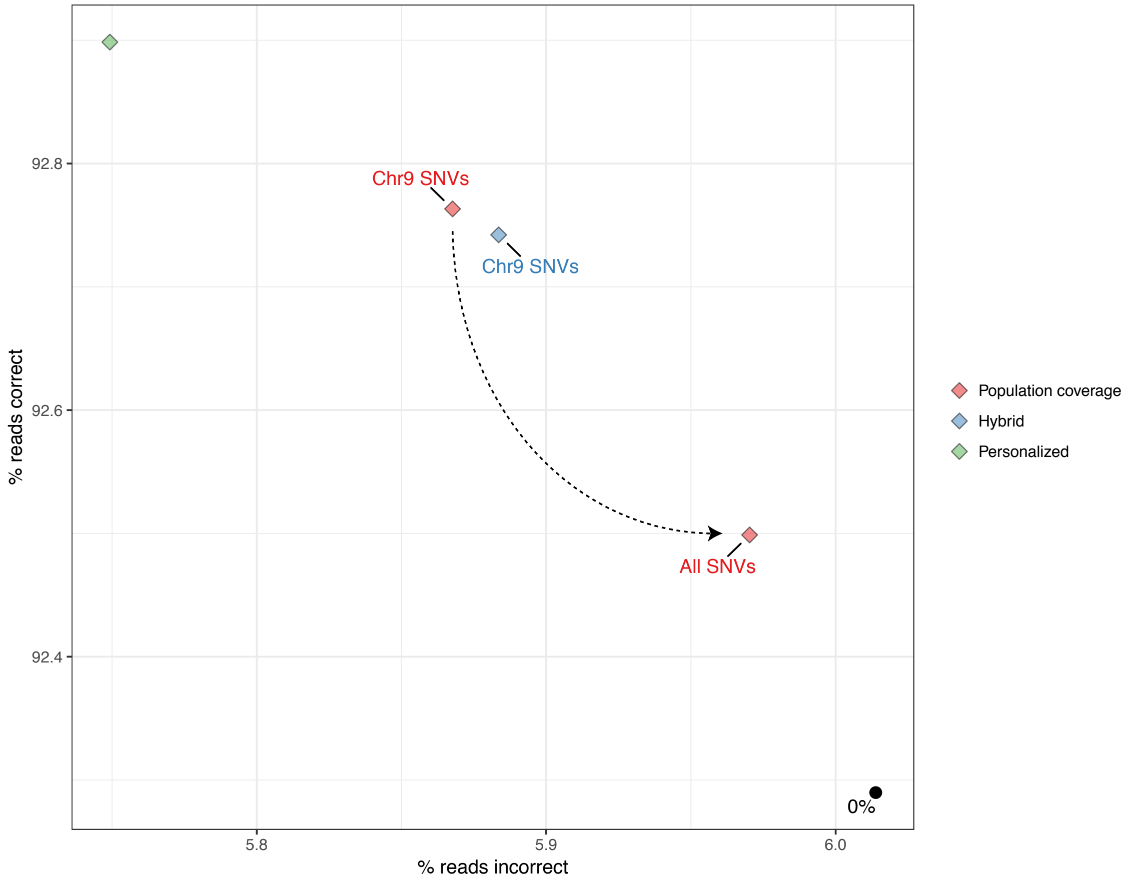

Fig S7: Effect of increasing graph genome complexity on accuracy. Blue and red diamonds mark the peak accuracy achieved by the *Pop Cov* and *Hybrid* models in two scenarios. In the first, we aligned the set of NA12878 simulated chromosome-9 reads to a whole-genome graph but containing only the optimal set of SNVs from chromosome 9 (“Chr 9 SNVs”). In the second, we aligned to a whole-genome graph with SNVs from across the entire genome (“All SNVs”). The added complexity from the non-chromosome-9 variants drove peak accuracy farther away from the personalized-genome ideal (dotted line), where the personalized ideal was customized to NA12878.

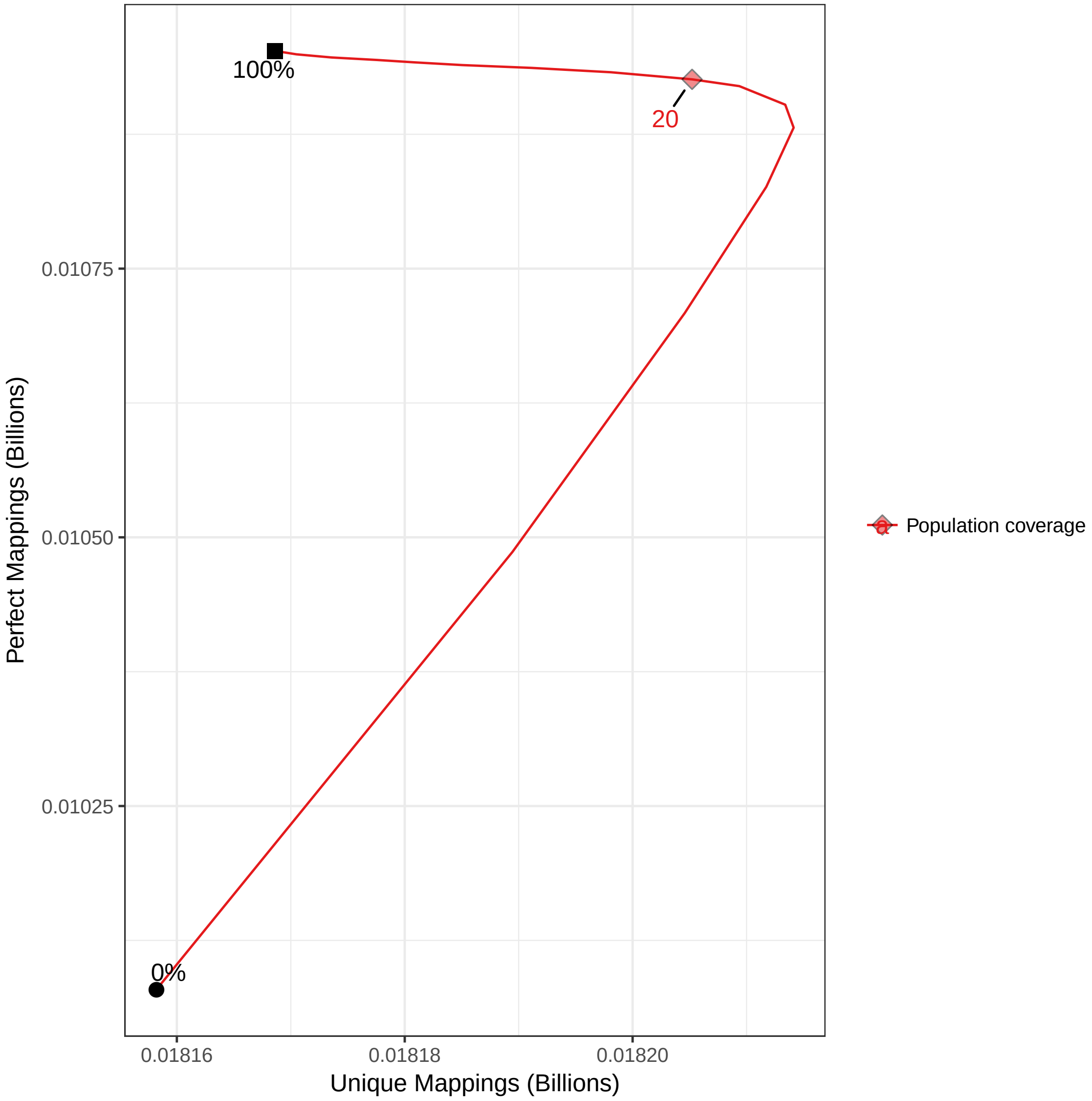

Fig S8: Unique/perfect plot for reads simulated from chromosome 9. This is the same set of input reads and the same reference genomes and indexes as are evaluated in Figure 1 in the main text. While the maximal correct-minus-incorrect measure is reached at 8% of SNVs (Figure 1c), the maximal unique-plus-perfect measure peaks at 20% of SNVs. So while the correct-minus-incorrect and unique-plus-perfect measures both attempt to capture the variant-inclusion trade-off, they do not peak at the same fraction of SNVs.

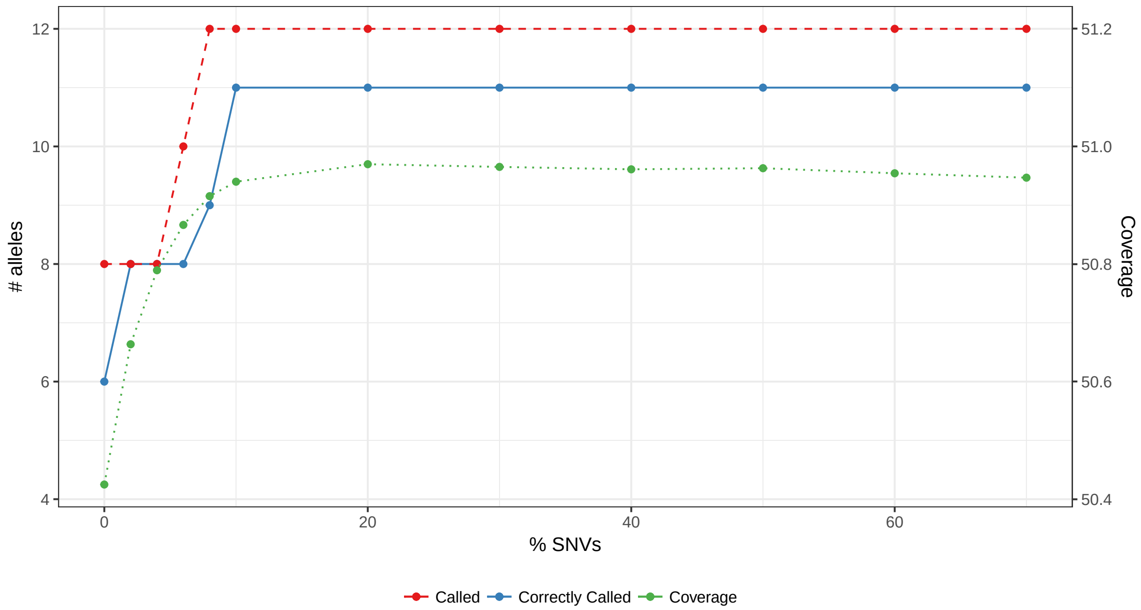

Fig S9: Improvement in HLA typing recall and accuracy when including more variants in the augmented reference. Kourami only makes a call for an allele when there is sufficient evidence, so we distinguish between the number of calls made by Kourami (red) and the number of correct calls (blue). There are 6 diploid genes, making 12 alleles the maximum callable. Including more variants in the reference quickly and substantially improves HLA typing recall, with the number of correctly called alleles plateauing at 10% of variants included, consistent with other results presented here. One of the calls is consistently incorrect.

References

[1] R. V. Satya, N. Zavaljevski, and J. Reifman. "A new strategy to reduce allelic bias in RNA-Seq readmapping". In: *Nucleic Acids Res.* 40.16 (Sept. 2012), e127.

[2] J. Sirén, N. Välimäki, and V. Mäkinen. "Indexing graphs for path queries with applications in genome research". In: *IEEE/ACM Transactions on Computational Biology and Bioinformatics* 11.2 (2014), pp. 375–388.

[3] L. Huang, V. Popic, and S. Batzoglou. "Short read alignment with populations of genomes". In: *Bioinformatics* 29.13 (July 2013), pp. i361–370.

[4] A. Auton, L. D. Brooks, R. M. Durbin, E. P. Garrison, H. M. Kang, J. O. Korbel, J. L. Marchini, S. McCarthy, G. A. McVean, G. R. Abecasis, et al. "A global reference for human genetic variation". In: *Nature* 526.7571 (Oct. 2015), pp. 68–74.

[5] Kim D. and Salzberg S.L. *HISAT2*. `https://github.com/infphilo/hisat2`. [Online; accessed 15-May-2017]. 2017.

[6] H. Lee and C. Kingsford. "Kourami: graph-guided assembly for novel human leukocyte antigen allele discovery". In: *Genome Biol.* 19.1 (Feb. 2018), p. 16.

[7] H. Li. *Aligning sequence reads, clone sequences and assembly contigs with BWA-MEM*. 2013. eprint: `arXiv:1303.3997`.

[8] A. T. Dilthey, P. A. Gourraud, A. J. Mentzer, N. Cereb, Z. Iqbal, and G. McVean. "High-Accuracy HLA Type Inference from Whole-Genome Sequencing Data Using Population Reference Graphs". In: *PLoS Comput. Biol.* 12.10 (Oct. 2016), e1005151.
